# Emerging insights into the role of BDH1 in the pathogenesis of human cancer

**DOI:** 10.3389/fonc.2026.1785200

**Published:** 2026-06-03

**Authors:** Penghui Wang, Yunmeng Yan, Zhengrong Liu, Guoxun Feng, Wei Yu

**Affiliations:** 1 Department of General Surgery, Beijing Tiantan Hospital, Capital Medical University, Beijing, China; 2 Key Clinical Laboratory of Henan Province, Department of Clinical Laboratory, The First Affiliated Hospital of Zhengzhou University, Zhengzhou, China

**Keywords:** BDH1, cancer, ketone body metabolism, metabolic reprogramming, pathogenesis

## Abstract

β-Hydroxybutyrate dehydrogenase 1 (BDH1) encodes a key enzyme in the metabolism of ketone bodies and plays a critical role in maintaining cellular energy homeostasis. As a member of the short-chain dehydrogenase/reductase (SDR) family, BDH1 catalyzes the reversible conversion of β-hydroxybutyrate (BHB) to acetoacetate (AcAc), a process that is particularly vital during states of metabolic stress, such as fasting or prolonged exercise. Beyond normal physiology, BDH1 functions as a context-dependent metabolic rheostat in human cancers, supporting altered neoplastic energy metabolism and conferring stress resistance. Its dysregulation is associated with tumor microenvironment adaptation, malignant progression, and differential therapeutic responses, positioning BDH1 as both a potential prognostic biomarker and a promising therapeutic target in precision oncology.

## Introduction

Cancer remains a major public health challenge worldwide. In the United States alone, more than 2 million new cancer cases and more than 600,000 cancer deaths are projected to occur in 2026 (1). Despite continuous advancements in treatment that have significantly improved survival rates for many malignancies, the overall burden of cancer underscores the urgent need for novel therapeutic targets (1). Cancer cells exhibit remarkable metabolic plasticity that allows them to adapt to changing environmental conditions and sustain uncontrolled proliferation. Understanding how cancers interact with host metabolism and how environmental and physiological parameters influence tumor progression is essential for improving cancer treatment (2, 3). This metabolic remodeling frequently involves coopting existing metabolic infrastructure to sustain rapid tumor progression and adapt to harsh tumor microenvironments (4).

β-Hydroxybutyrate dehydrogenase 1 (BDH1) is a mitochondrial enzyme that has sparked recent interest as a metabolic target in diverse malignancies due to its reported involvement in tumor survival, metastasis, and immune evasion (5–7). In both normal and cancer cells, BDH1 plays an important role in ketolysis, catalyzing the reversible conversion of β-hydroxybutyrate (BHB) to acetoacetate (AcAc), and linking ketone body metabolism to mitochondrial respiration (6) and redox regulation (7–10). Recent studies have implicated BDH1 in modulation of oxidative stress and tumor microenvironment adaptations across various malignancies, correlating its altered expression and dysregulation with aggressive tumor phenotypes, poor outcomes, and differential therapeutic responses (5, 11–15). These findings suggest complex, context-dependent roles for BDH1 in tumorigenesis.

This narrative review proposes that BDH1-mediated ketone body metabolism acts as a context-dependent metabolic rheostat that influences tumor energy production, epigenetic reprogramming, and microenvironment adaptation. By systematically summarizing the expression patterns and functional roles of BDH1, whether acting as an oncogene or a tumor suppressor, across different cancer types, this review aims to elucidate its potential as a prognostic biomarker and to discuss implications for targeted therapeutic strategies, including ketogenic diets and mitochondrial-targeted therapies.

## Structure and normal cell functions of BDH1

**Figure 1:**
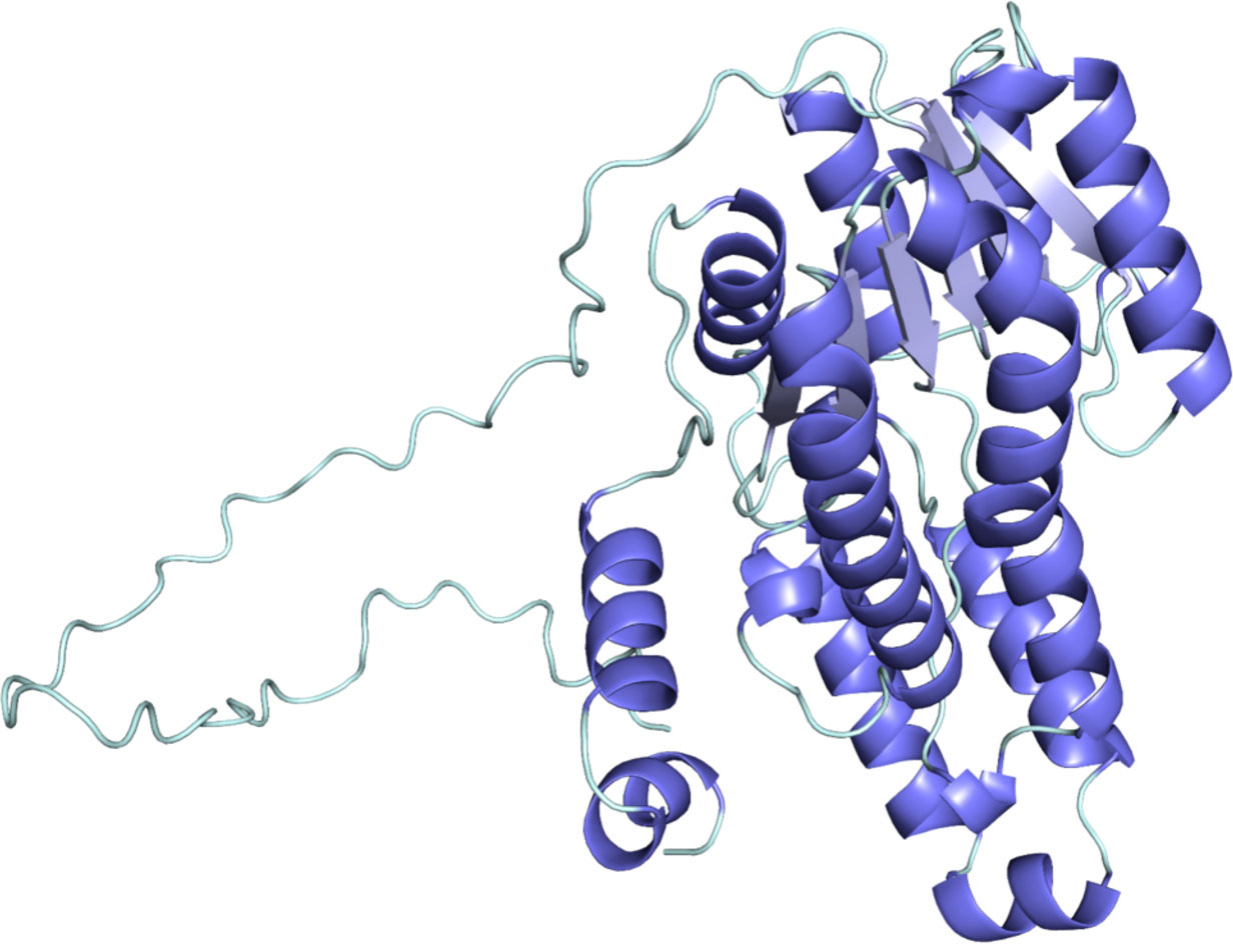
3D structure of BDH1 gene. Alphafold3 was used to predict the 3D structure of the protein.

BDH1, also referred to as BDH or SDR9C1, is part of the gene family that encodes short-chain dehydrogenase/reductase (SDR) enzymes (16–18). Churchill et al. (1992) conducted the first analysis of the primary structure of BDH1 in rat liver using a combination of cDNA clone nucleotide sequencing and purified protein amino acid sequencing: the BDH1 gene comprises 1,435 bases and encodes both the whole amino acid sequence of mature BDH and the primary peptide of precursor BDH; hybridization analysis of poly(A+) rat liver mRNA showed two bands (about 3.2 kb and 1.7 kb in size); comparative analysis of the BDH1 amino acid sequence with other reported sequences revealed a high degree of homology with the SDR superfamily. In 1992, Fleischer et al. cloned the BDH1 gene in a human heart and predicted that the gene consists of 297 amino acids and does not contain any transmembrane helix (19). According to NCBI data, the human BDH1 gene is located on chromosome 3, comprises 11 exons, and encodes a total of 343 amino acids. The structure of BDH1 is displayed in **Figure 1**. In recent years, this gene has been extensively studied in humans, rats, mice, rabbits, and other organisms.

### Catalysis and metabolism

BDH1 catalyzes the reversible, NADH/NAD^+^-coupled interconversion of β-hydroxybutyrate (BHB) and acetoacetate (AcAc) (6, 7). This near-equilibrium reaction is essential for both hepatic ketogenesis, where AcAc is reduced to BHB for export, and extrahepatic ketolysis, where BHB is oxidized back to AcAc for energy production. The enzyme requires phosphatidylcholine for optimal catalytic activity, and its homotetrameric quaternary structure allows cooperative substrate binding. The active site contains conserved catalytic residues His146 and Ser138, which are critical for the dehydrogenase reaction mechanism (19).

**Figure 2:**
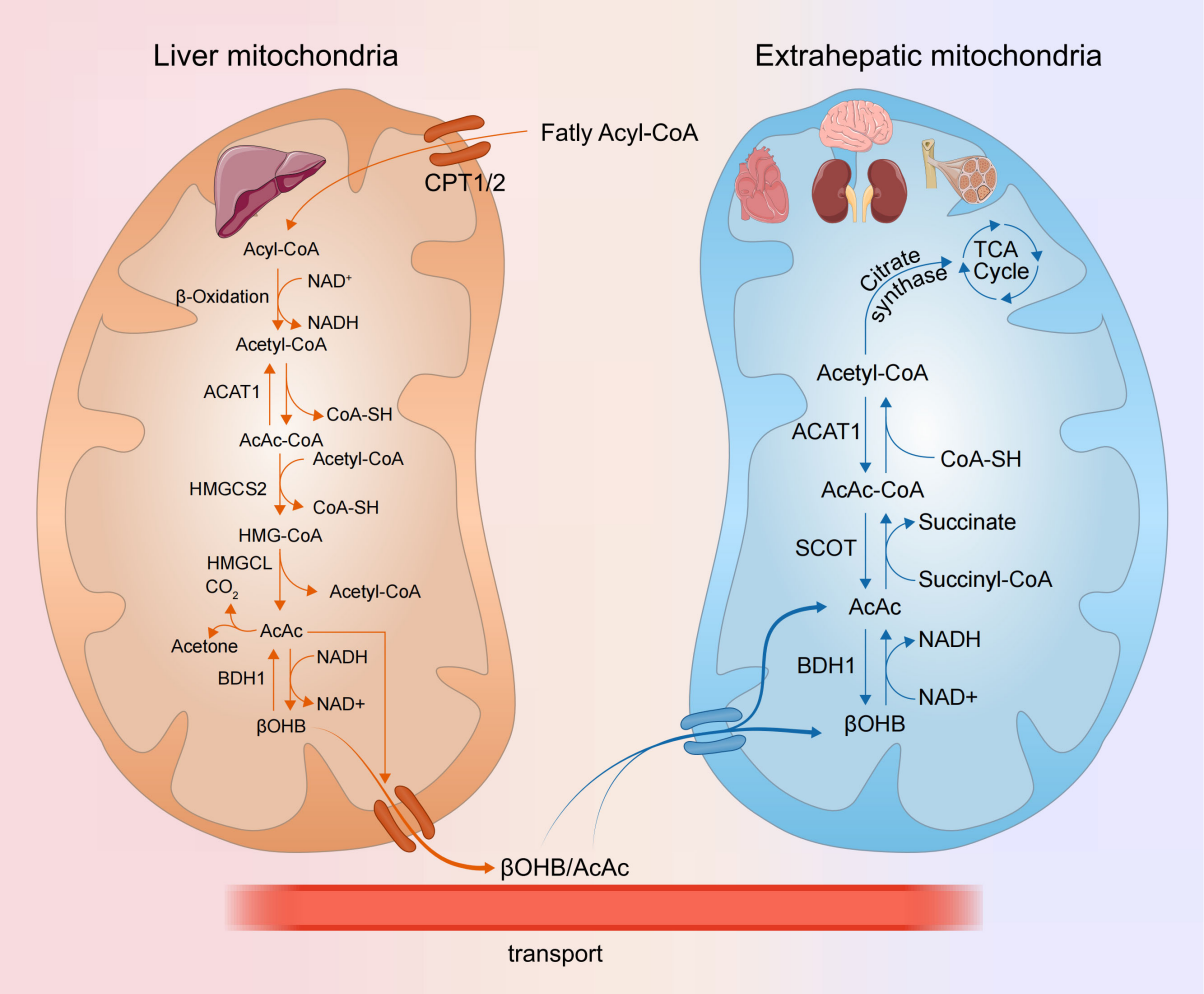
Metabolic functions of BDH1 in hepatic ketogenesis and extrahepatic ketolysis. BHB, β-hydroxybutyrate; AcAc, acetoacetate.

**Figure 2** integrates the major metabolic functions of BDH1 in ketogenesis and ketolysis. Energy programmability is a hallmark of cancer, with dysregulated metabolic processes such as aerobic glycolysis, fatty acid metabolism, and amino acid metabolism implicated in various cancers (20–22). Among these pathways, ketone metabolism is a lipid-derived metabolic route coordinated by 3-hydroxymethylglutaryl-CoA synthase (HMGCS2), hydroxymethylglutaryl coenzyme A lyase (HMGCL), 3-oxoacid CoA transferase 1 (OXCT1), and BDH1. During ketogenesis, HMGCS2 catalyzes the condensation of acetyl-CoA and AcAc-CoA to generate hydroxymethylglutaryl (HMG)-CoA; HMGCL then cleaves HMG-CoA to produce acetyl-CoA and AcAc; and AcAc is subsequently converted into BHB by BDH1 through an NADH/NAD^+^-coupled near-equilibrium reaction. During ketolysis, extrahepatic mitochondrial BDH1 catalyzes the first step by converting BHB back to AcAc; OXCT1 converts AcAc to AcAc-CoA; and thiolase produces acetyl-CoA, which enters the tricarboxylic acid (TCA) cycle for ATP production (7) as shown in **Figure 2**. This complete depiction of ketone-body flux clarifies why co-option of BDH1-mediated metabolism can be important for cancer cells under nutrient stress (4, 20–22).

### Deficiencies and disease

Under conditions of unrestricted food access, BDH1-deficient mice exhibit normal growth; however, fasting leads to significant differences compared to controls: systemic BDH1 deficiency results in a marked increase in the AcAc/BHB ratio and hepatic steatosis during fasting, highlighting the importance of ketogenesis in maintaining lipid energy balance in the liver (23).

The expression of BDH1 is significantly reduced in the livers of metabolic-associated fatty liver disease patients. Xu et al. (2022) found a significant reduction in BDH1 expression in the kidneys of diabetic kidney disease (DKD) mouse models, as well as in diabetic patients. Consistent with these findings, decreased BDH1 expression was also observed in high glucose- or palmitic acid-treated human renal tubular epithelial (HK-2) cells (8).

Knockdown of BDH1 leads to excessive production of reactive oxygen species (ROS), triggering ROS-induced inflammation and apoptosis in both liver and HK-2 cells. This ROS overproduction occurs because BDH1 knockdown disrupts the BHB-AcAc-succinate-fumarate metabolic flux, leading to impaired NRF2 (Nuclear Factor Erythroid 2-Related Factor 2) activation. NRF2 is a master regulator of the cellular antioxidant defense system; its reduced activation results in decreased expression of downstream antioxidant enzymes (e.g., SOD, catalase, HO-1), thereby leading to ROS accumulation. Additionally, impaired ketone body metabolism reduces the availability of NADH for mitochondrial complex I, potentially increasing electron leakage and superoxide production.

BDH1 upregulation protects liver cells from lipotoxicity and glucotoxicity by suppressing ROS accumulation via NRF2 activation driven by enhanced BHB-AcAc-succinate-fumarate metabolic flux. Notably, adeno-associated virus (AAV)-mediated overexpression of BDH1 effectively improved hepatic function markers, reduced inflammation and fibrosis, and attenuated apoptosis in fatty liver and kidney tissues in mice (8, 9). The mechanism underlying these protective effects involves enhanced BHB-to-AcAc conversion, which increases succinate and fumarate levels in the TCA cycle. Fumarate, as a potent activator of NRF2, promotes the transcription of antioxidant genes, thereby reducing ROS-mediated NF-κB activation and downstream inflammatory cytokine production (e.g., TNF-α, IL-6). The suppression of oxidative stress and inflammation consequently attenuates fibrogenic signaling pathways (e.g., TGF-β/Smad), reducing collagen deposition and fibrosis.

Cardiac-specific upregulation of BDH1 enhances ketone body utilization and confers resistance to pressure overload-induced pathological cardiac remodeling (10). This cardioprotective effect is attributed to enhanced ketone body utilization efficiency, which provides an alternative energy source to the failing heart where glucose and fatty acid oxidation may be compromised. The increased BHB metabolism activates antioxidant defense genes, reducing pressure overload-induced oxidative damage. Furthermore, efficient ketone body utilization improves mitochondrial energetics and reduces the ATP deficit associated with pathological cardiac remodeling, thereby conferring resistance to pressure overload. These findings collectively position BDH1 as a multifaceted gene with critical roles spanning metabolism, aging, and neurodevelopmental and psychiatric disorders.

### BDH1 in cancer: context-dependent roles

The metabolic reprogramming of cancer cells frequently involves the co-option of alternative fuel sources such as ketone bodies to sustain proliferation, survival, and adaptation under nutrient stress (3, 4, 7). BDH1 occupies a central position in this process because it links ketone body oxidation to mitochondrial respiration, the NAD^+^/NADH redox balance, oxidative stress responses, and BHB-related epigenetic regulation. Across human malignancies, BDH1 expression varies markedly in a tissue-dependent manner, supporting malignant growth in ovarian, pancreatic, lung, and prostate cancers, and is associated with tumor-promoting phenotypes in liver cancer and acute myeloid leukemia when BDH1 expression is reduced (5, 11–15, 24, 25). These distinct and context-dependent roles in individual cancers are detailed in the subsections below and summarized in **Table 1**.

**Table 1:** Context-dependent expression, activity, and clinical implications of BDH1 across human cancers.

Cancer type	Context	Mutation type	Experimental categories	Clinical impact of BDH1	BDH1 activity data	References
33 human cancer cell lines	Expressed in all lines	Variable expression	HeLa, PANC-1 cell lines, mouse xenograft models	Regulation of ketone body metabolism, impacts tumor sensitivity to ketogenic diets.	Functional ketolysis confirmed in high-expressing lines (HeLa); low-expressing lines (PANC-1) show minimal ketone utilization	(44)
Glioblastoma	Reduced BDH1 Expression	Downregulation	Glioblastoma cell lines, patient samples, mouse studies	Poor prognosis. AcAc induces apoptosis in glioma cells.	Reduced ketone body oxidation capacity; direct activity assays not reported	(29, 30)
Breast cancer	Unique BDH1 Variant Found	Non-synonymous variant	MCF7 cell line, patient-derived samples	BDH1 upregulated in tumor stroma, promoting ketone body metabolism.	Stromal ketogenesis fuels epithelial OXPHOS; direct BDH1 activity not measured	(32, 33)
Prostate cancer	Upregulated BDH1	Elevated transcript and protein	LNCaP and LNCaP-SF cell lines; patient specimens	Associated with tumor progression, castration resistance.	Elevated protein levels suggest increased ketolytic flux; direct enzymatic assays needed	(11, 37)
AML	Low BDH1 Expression	Downregulation at mRNA level	Patient samples	BDH1 downregulation promotes AML cell proliferation.	Transcriptional suppression implies reduced activity; not directly measured	(12)
Liver cancer	Altered BDH1 Expression	Downregulation at mRNA and protein level	Patient samples and liver cancer cells	Low BDH1 expression linked to poor prognosis.	Serum starvation reactivates ketolysis in HCC cells; activity correlates with proliferation under nutrient stress	(5, 13)
Lung cancer	Elevated BDH1 Levels	Upregulation at mRNA and protein level	Patient samples, xenograft models	BDH1 regulates autophagy via AMPK-mTOR; H3K9bhb modifications.	Enhanced Kbhb levels indicate increased catalytic activity; pimozide/crizotinib inhibit BDH1 activity	(14, 15)
OSCC	Downregulated BDH1	Downregulation at mRNA level	Patient samples, GEO database	BDH1 downregulation correlates with tumor progression.	Correlated with mitochondrial activity; direct activity not measured	(47, 48)

### Ovarian and pancreatic cancer

Ovarian cancer is a highly lethal gynecological malignancy, and recent studies have begun to uncover the role of BDH1 in its progression. Sun et al. demonstrated that BDH1 acts as an oncogene in ovarian cancer by driving tumor progression, regulating cell cycling, and maintaining stemness through activation of the Wnt/β-catenin signaling pathway (24). Furthermore, BDH1 has been incorporated into a prognostic signature based on lactate metabolism-related genes for serous ovarian cancer, highlighting its potential clinical relevance in predicting patient outcomes (26). Together, these data support the oncogenic arm of the BDH1 model, in which increased BDH1 activity may reinforce proliferative signaling and metabolic adaptability in ovarian cancer (24, 26).

In pancreatic cancer, BDH1 also appears to promote malignant phenotypes. Wei et al. reported that BDH1 enhances pancreatic cancer cell proliferation by regulating the NAD^+^/NADH balance and modulating mitochondrial acetylation (25). Additionally, ketone body metabolism reprogramming, involving enzymes such as BDH1, has been shown to diminish pancreatic cancer cachexia, suggesting a complex interplay between tumor metabolism and systemic host responses (27). These findings link BDH1 to both cell-intrinsic redox control and host-tumor metabolic exchange, supporting its inclusion in the tumor-promoting side of the context-dependent model (25, 27).

### Glioblastoma

Glioblastoma is the most aggressive primary brain tumor in both children and adults, causing significant mortality annually in the United States (1, 28). The key ketone body metabolic enzymes BDH1, OXCT1, and ACAT1 are expressed at both the mRNA and protein levels in glioma cell lines (29). Graham et al. revealed that these enzymes are dramatically decreased in adult and pediatric glioblastoma, and hypoxic conditions further reduced their mRNA expression levels (30). These observations suggest that glioblastoma may have limited capacity for complete ketone body oxidation compared with tumors that retain high BDH1 expression, providing a mechanistic basis for its distinct response to ketogenic interventions (29, 30).

*In vivo* studies showed that a ketogenic diet significantly elevated blood BHB levels without altering blood glucose levels or improving survival outcomes (30). While BHB protected hippocampal neurons from glucose withdrawal-induced cell death, it failed to safeguard glioma cell lines. Treatment of glioblastoma cell lines with the glycolytic inhibitor 2-deoxy-D-glucose and the ketone body AcAc, but not BHB, led to dose-dependent reductions in viability. Western blot analysis confirmed that AcAc caused apoptosis and reduced neurosphere formation, while co-treatment led to greater cell death than either treatment alone. Mechanistically, AcAc directly enhances mitochondrial uncoupling protein 2 (UCP2), potentially explaining its synergistic effects through multifaceted inhibition of glycolytic metabolism (29, 30). Thus, the glioblastoma literature supports a model in which ketone body effects depend on the specific ketone metabolite, tumor ketolytic capacity, and the integrity of downstream mitochondrial metabolism (29, 30).

### Breast cancer

Breast cancer is the most prevalent cancer among females worldwide, and it remains a leading cause of cancer-related mortality in women globally (1, 31). Utilizing novel splice-aware alignment algorithms, Kang et al. identified a unique non-synonymous RNA variant of BDH1 specifically associated with breast cancer tissues (32).

**Figure 3:**
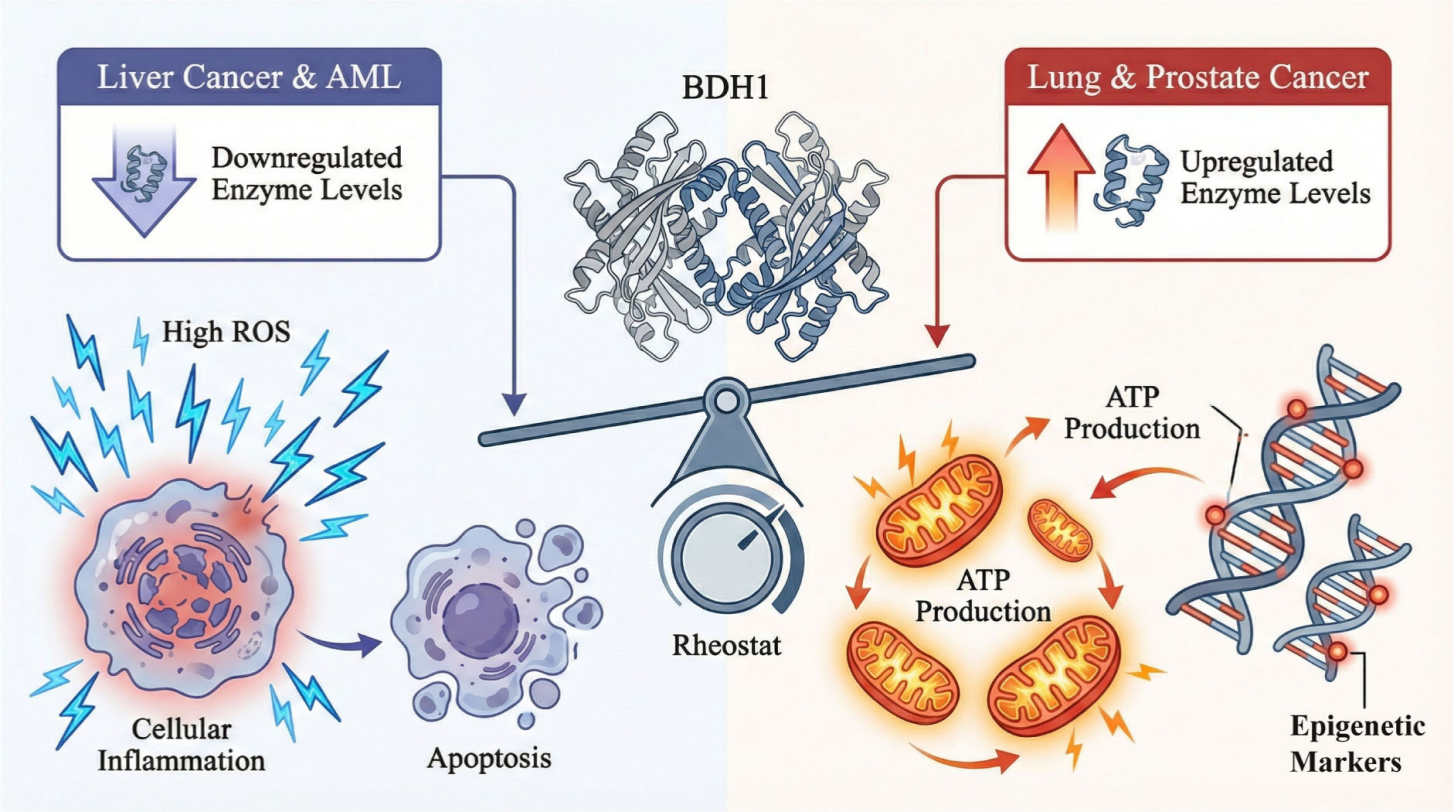
The “Two-Compartment Tumor Metabolism” model in breast cancer.

Sotgia et al. found that cancer-associated fibroblasts (CAFs) exhibit a significant upregulation of enzymes required for ketone body production, including BDH1, driven by stromal caveolin-1 expression and/or serum starvation (33). Treatment with ketone bodies alone was sufficient to induce mitochondrial biogenesis in breast cancer cells. Analysis of human breast cancer specimens revealed distinct metabolic compartmentalization: enzymes involved in ketone body production (HMGCS2, HMGCL, BDH1) were predominantly expressed in the tumor-associated stroma, while enzymes linked to ketone body utilization, such as ACAT1, were primarily expressed in epithelial tumor cells. These findings support the “two-compartment tumor metabolism” model, in which stromal cells generate energy-rich metabolites such as ketone bodies that are transferred to and oxidized by epithelial cancer cells to fuel mitochondrial biogenesis and oxidative phosphorylation (33) (**Figure 3**). This stromal-epithelial division of ketone production and utilization provides a concrete example of how BDH1-associated metabolism can support tumor growth without requiring identical BDH1 functions in every cellular compartment (33).

### Prostate cancer

Prostate cancer is the most common cancer among men in the United States and encompasses clinically heterogeneous disease ranging from localized hormone-sensitive tumors to advanced castration-resistant prostate cancer (34–36). In this context, BDH1 appears to support malignant progression by providing metabolic flexibility. Among ketogenesis-related enzymes, BDH1 protein was markedly upregulated in androgen-independent LNCaP-SF cells, and complementary analyses confirmed significantly elevated BDH1 transcript and protein levels in high-grade tumors (37). Labanca et al. from the Gueron group further revealed dysregulation of ketolytic enzymes, including BDH1, during the transition to castration-resistant prostate cancer; BDH1 upregulation was associated with shorter time to biochemical recurrence and reduced progression-free survival (11). Collectively, these studies suggest that BDH1 contributes to tumor progression and resistance mechanisms, particularly in advanced and castration-resistant disease states (11, 34–37). These clinical and proteomic observations support classification of prostate cancer within the BDH1-high, ketolysis-associated arm of the proposed cancer model (11, 37).

### Acute myeloid leukemia

Analogous to its role in liver cancer, BDH1 exhibits tumor-suppressive functions in acute myeloid leukemia (AML), a hematological malignancy with a substantial global burden, particularly in women of child-bearing age (38). Jiang et al. investigated the expression of 10 key genes in ketone metabolism in AML blasts and found that BDH1 expression was significantly lower in AML blasts than in normal hematopoietic stem cells (12). Analyses of TCGA demonstrated a strong correlation between reduced BDH1 expression and poorer clinical outcomes. Functionally, reintroduction of BDH1 expression significantly inhibited viability and proliferation, while suppression promoted leukemic cell growth. These findings position BDH1 as a potential suppressor of AML progression and support the model that loss of BDH1-mediated metabolic control can promote malignant survival in selected cancer contexts (12).

### Liver cancer

BDH1 frequently serves as a tumor suppressor in liver cancer, a disease with a growing global burden associated with metabolic dysfunction (39, 40). In hepatocellular carcinoma, BDH1 is significantly downregulated and has been identified as an independent prognostic marker; reduced BDH1 expression correlates with adverse clinicopathological parameters and poorer outcomes (5, 41). Conversely, under nutrient deprivation, liver cancer cells can redirect metabolism toward ketolysis, indicating that the biological consequences of BDH1 dysregulation depend on the metabolic state of the tumor microenvironment (13).

Mechanistically, BDH1-associated metabolic alterations intersect with MYC and Wnt/β-catenin signaling pathways and may influence cancer stem cell phenotypes (5, 13). In addition, BDH1-related changes in BHB metabolism can affect histone H3 lysine 9 β-hydroxybutyrylation (H3K9bhb), an epigenetic acylation mark linked to BHB-derived metabolic signaling (15, 42). These findings indicate that BDH1 may regulate liver cancer biology through both metabolic and epigenetic mechanisms. They also explain why liver cancer can occupy the tumor-suppressive arm of the model under baseline BDH1 downregulation, while retaining the ability to reactivate ketolysis under nutrient deprivation (5, 13, 42).

### Lung cancer

In lung cancer, BDH1 is upregulated and acts as a key regulator of tumor progression and metastasis. Lung cancer continues to be the leading cause of cancer mortality worldwide, projected to cause more deaths in 2026 than colorectal and pancreatic cancers combined (1, 43). BDH1’s oncogenic role in lung cancer is characterized by enhanced ketolysis that provides additional acetyl-CoA for the TCA cycle and by β-hydroxybutyrylation-associated epigenetic regulation of downstream oncogenic targets (14, 15, 42).

BDH1 is significantly upregulated in lung cancer at the mRNA and protein levels, and elevated BDH1 expression correlates with metastasis (14). Mechanistically, BDH1 regulates intracellular lysine beta-hydroxybutyrylation (Kbhb); BDH1 represses H3K9bhb modification at the transcription start site of LRRC31, inhibiting its expression and modulating lung cancer progression (15, 42).

Potential BDH1 inhibitors, including pimozide and crizotinib, exhibit synergistic inhibitory effects in lung cancer with high BDH1 expression (15). Niu et al. also revealed that the AMPK/mTOR signaling pathway plays a critical role in BDH1-induced autophagy, providing an additional mechanistic link between BDH1 activity, stress adaptation, and lung cancer progression (14). Together with the LRRC31/H3K9bhb axis, these findings support the model that BDH1 can promote lung cancer through both bioenergetic and epigenetic mechanisms (14, 15, 42).

### Ketogenic metabolism in cancer

Ketogenic metabolism provides a useful framework for understanding the proposed BDH1 rheostat model in cancer. In this model, BDH1 does not behave as a uniformly oncogenic or tumor-suppressive factor. Instead, its biological output depends on tumor lineage, substrate availability, NAD^+^/NADH balance, mitochondrial competence, and whether BHB is primarily used as an oxidative fuel, a redox regulator, or a donor for epigenetic β-hydroxybutyrylation (6, 7, 13, 15, 42, 44, 45). Thus, BDH1 may promote survival in tumors that efficiently oxidize ketone bodies, while reduced BDH1 activity may worsen oxidative stress and malignant signaling in other contexts.

Mao et al. evaluated BDH1 expression patterns across 33 human cancer cell lines: BDH1 was expressed in all lines, but expression levels varied dramatically. They selected two representative cell lines, HeLa with high BDH1 mRNA expression and PANC-1 with low BDH1 mRNA expression. In mice with HeLa xenografts, the ketogenic diet accelerated cancer growth and reduced survival time, likely because these tumors actively utilized ketone bodies as an energy source. Conversely, the ketogenic diet significantly inhibited the growth of PANC-1 xenograft tumors (44).

When BHB was introduced to cell cultures, it markedly stimulated the proliferation of HeLa cells, but not PANC-1 cells. Downregulation of BDH1 decreased the susceptibility of HeLa cells to the ketogenic diet both *in vitro* and *in vivo*. Notably, HeLa cells with high BDH1 expression actively metabolize ketone bodies through mitochondrial ketolysis. Under ketogenic diet conditions, their reliance on mitochondrial oxidative phosphorylation (OXPHOS) for energy production is enhanced, as ketone body-derived acetyl-CoA must enter the TCA cycle and electron transport chain (ETC) for ATP generation. This increased mitochondrial dependence may make these cells more susceptible to mitochondrial inhibitors, such as complex I inhibitors including metformin, because disrupting the electron transport chain would selectively deplete ATP supply in cells relying on ketolysis (44, 45). Future studies should directly test whether ketogenic diet-fed tumors with high BDH1 expression show enhanced sensitivity to mitochondrial-targeted therapies.

BDH1’s role in mediating ketogenic diet efficacy highlights the potential for stratifying patients by BDH1 levels in clinical applications. However, BDH1 protein levels do not always correlate with increased enzymatic activity or metabolic flux (45), as factors such as post-translational modifications, cofactor availability (e.g., NAD^+^/NADH ratio, phosphatidylcholine), and allosteric regulation can independently modulate BDH1 activity. Therefore, patient stratification for ketogenic diet therapy should ideally incorporate both BDH1 expression levels and direct enzymatic activity measurements to ensure reliable clinical predictions.

### Oral squamous cell carcinoma

Oral squamous cell carcinoma (OSCC) contributes significantly to the global burden of lip and oral cavity cancers, particularly among adolescents and young adults (46). While significant differences in BDH1 expression between tumor and pre-tumor tissues in OSCC have not been identified, BDH1 mRNA expression levels have been linked to mitochondrial activity and content (47). BDH1 is downregulated in OSCC, and piR-37620, piR-52205, and piR-57816 were predicted to target BDH1 by binding to its 3′UTR regions (48). These findings suggest that BDH1 may be connected to mitochondrial remodeling in OSCC, but direct enzymatic activity studies are still needed before OSCC can be assigned confidently to either the oncogenic or tumor-suppressive arm of the model (47, 48).

### Therapeutic implications: targeting BDH1 and ketone metabolism in cancer

**Figure 4:**
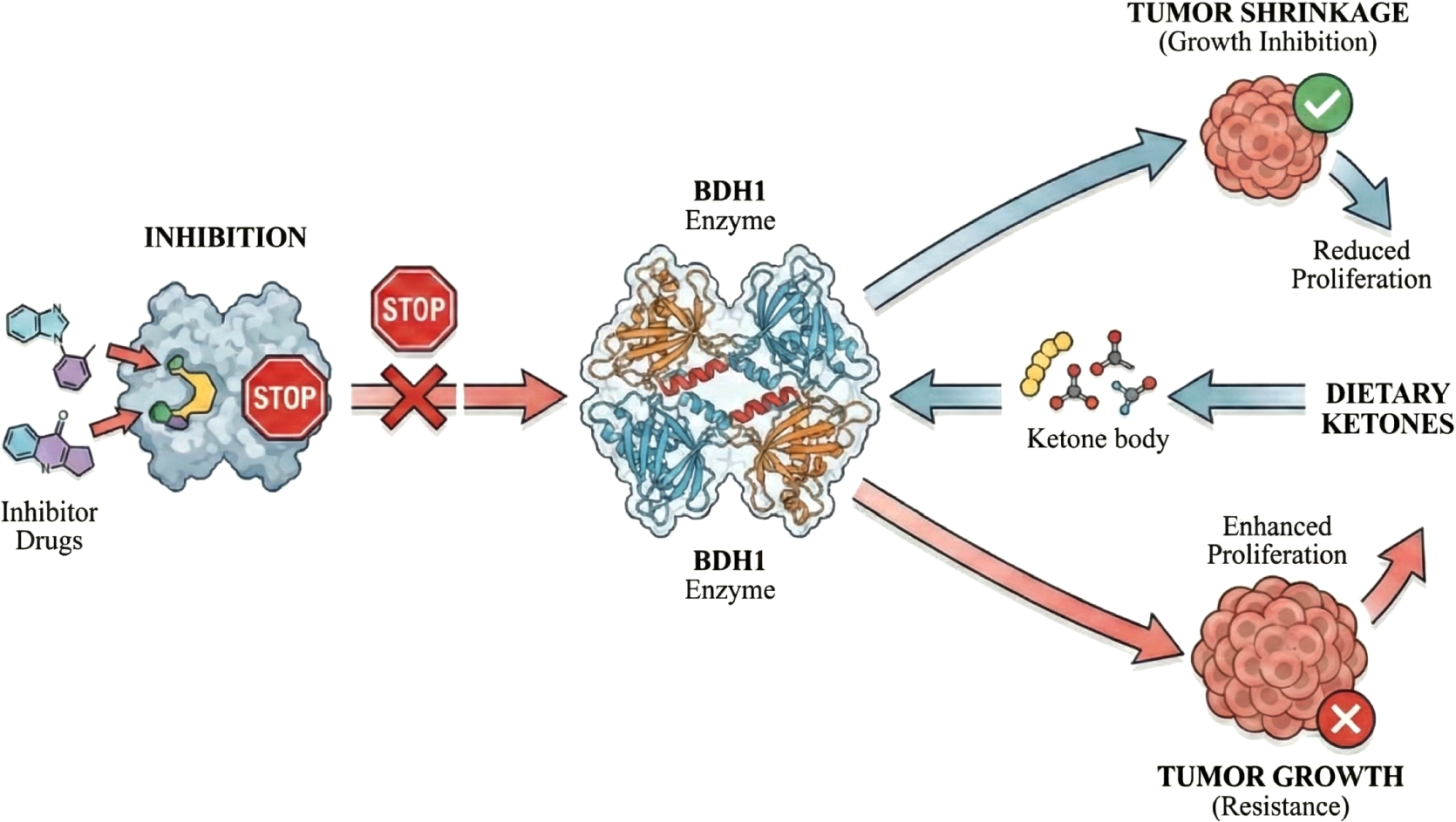
The double-edged sword role of BDH1 in cancer pathogenesis.

The emerging understanding of BDH1’s role in cancer metabolism opens multiple avenues for therapeutic intervention. BDH1 acts as a metabolic rheostat with distinct functions depending on the tissue context in the proposed model (**Figure 4**), in which liver cancer and AML represent settings where BDH1 downregulation may promote oxidative stress, inflammatory signaling, and malignant survival pathways, whereas lung and prostate cancers represent settings where BDH1 upregulation promotes ketolysis for energy production and facilitates epigenetic reprogramming through H3K9bhb-related mechanisms (**Table 1**).

Therapeutically, the most defensible use of BDH1 biology is patient stratification. Tumors with high BDH1 expression and intact ketolytic machinery may be more capable of oxidizing ketone bodies, as suggested by the divergent HeLa and PANC-1 xenograft responses to a ketogenic diet (44). In these tumors, combining dietary or metabolic interventions with mitochondrial inhibitors may be more rational than applying ketogenic strategies broadly (44, 45). Conversely, in contexts in which BDH1 is suppressed and oxidative stress is increased, such as liver cancer and AML, strategies that restore antioxidant metabolic flux may be more relevant than BDH1 inhibition (5, 12, 13).

Direct pharmacological targeting also warrants study. Pimozide and crizotinib have been reported to inhibit BDH1-associated activity in lung cancer with high BDH1 expression (15), and BDH1-induced autophagy through AMPK/mTOR signaling provides an additional axis for combination therapy (14). Because BDH1 protein abundance may not always reflect catalytic flux, future trials should measure BDH1 expression together with enzymatic activity, NAD^+^/NADH status, Kbhb/H3K9bhb marks, and ketolytic enzymes such as OXCT1 and ACAT1 (6, 7, 14, 15, 42, 45).

## Conclusion

In conclusion, BDH1 is a complex mitochondrial enzyme that bridges fundamental ketone body metabolism with cancer biology. Under normal conditions, its catalytic conversion of BHB and AcAc plays essential roles in energy homeostasis and antioxidant defense. In neoplastic settings, however, this metabolic function can be exploited for advantageous energy production and tumor microenvironment adaptation. Depending on tissue context, substrate availability, redox state, and epigenetic output, BDH1 acts as a metabolic rheostat-either functioning as a tumor suppressor, as observed in liver cancer and AML, where its loss promotes oxidative stress and malignant survival signaling, or as an oncogene, as observed in lung and prostate cancers, where its upregulation fuels proliferation and enables epigenetic reprogramming. Recognizing these context-dependent roles provides a biological basis for utilizing BDH1 expression and enzymatic activity as predictive biomarkers, while also highlighting the need for tailored therapeutic strategies such as specific BDH1 inhibitors, mitochondrial-targeted therapies, or carefully stratified ketogenic dietary interventions in precision oncology.

## References

[1] Siegel RL Kratzer TB Wagle NS Sung H Jemal A . Cancer statistics, 2026. CA Cancer J Clin. (2026) 76:e70043. doi: 10.3322/caac.70043. PMID: 41528114 PMC12798275

[2] Hanahan D Weinberg RA . Hallmarks of cancer: the next generation. Cell. (2011) 144:646–74. doi: 10.1016/j.cell.2011.02.013. PMID: 21376230

[3] Swanton C Bernard E Abbosh C André F Auwerx J Balmain A . Embracing cancer complexity: hallmarks of systemic disease. Cell. (2024) 187:1589–616. doi: 10.1016/j.cell.2024.02.009. PMID: 38552609 PMC12077170

[4] Yoshida GJ . Metabolic reprogramming: the emerging concept and associated therapeutic strategies. J Exp Clin Cancer Res. (2015) 34:111. doi: 10.1186/s13046-015-0221-y. PMID: 26445347 PMC4595070

[5] Luo W Wu S Zhang F Chen X Ma Y Mo Y . Decreased expression of 3-hydroxybutyrate dehydrogenase 1 is a prognostic marker and promotes tumor progression in hepatocellular carcinoma. Pathol Res Pract. (2022) 238:154111. doi: 10.1016/j.prp.2022.154111. PMID: 36115334

[6] Nelson AB Queathem ED Puchalska P Crawford PA . Metabolic messengers: ketone bodies. Nat Metab. (2023) 5:2062–74. doi: 10.1038/s42255-023-00935-3. PMID: 38092961

[7] Puchalska P Crawford PA . Multi-dimensional roles of ketone bodies in fuel metabolism, signaling, and therapeutics. Cell Metab. (2017) 25:262–84. doi: 10.1016/j.cmet.2016.12.022. PMID: 28178565 PMC5313038

[8] Wan SR Teng FY Fan W Xu BT Li XY Tan XZ . BDH1-mediated βoHB metabolism ameliorates diabetic kidney disease by activation of NRF2-mediated antioxidative pathway. Aging (Albany Ny). (2023) 15:13384–410. doi: 10.18632/aging.205248. PMID: 38015723 PMC11622694

[9] Xu BT Teng FY Wu Q Wan SR Li XY Tan XZ . BDH1 overexpression ameliorates hepatic injury by activation of Nrf2 in a MAFLD mouse model. Cell Death Discov. (2022) 8:49. doi: 10.1038/s41420-022-00840-w. PMID: 35115498 PMC8814004

[10] Uchihashi M Hoshino A Okawa Y Ariyoshi M Kaimoto S Tateishi S . Cardiac-specific BDH1 overexpression ameliorates oxidative stress and cardiac remodeling in pressure overload-induced heart failure. Circ Heart Fail. (2017) 10(12):e004417. doi: 10.1161/circheartfailure.117.004417. PMID: 29242353

[11] Labanca E Bizzotto J Sanchis P Anselmino N Yang J Shepherd PDA . Prostate cancer castrate resistant progression usage of non-canonical androgen receptor signaling and ketone body fuel. Oncogene. (2021) 40:6284–98. doi: 10.1038/s41388-021-02008-9. PMID: 34584218 PMC8566229

[12] Han F Zhao H Lu J Yun W Yang L Lou Y . Anti-tumor effects of BDH1 in acute myeloid leukemia. Front Oncol. (2021) 11:694594. doi: 10.3389/fonc.2021.694594. PMID: 34150668 PMC8213090

[13] Huang D Li T Wang L Zhang L Yan R Li K . Hepatocellular carcinoma redirects to ketolysis for progression under nutrition deprivation stress. Cell Res. (2016) 26:1112–30. doi: 10.1038/cr.2016.109. PMID: 27644987 PMC5113304

[14] Zhang Z Bi X Lian X Niu Z . BDH1 promotes lung cancer cell proliferation and metastases by PARP1-mediated autophagy. J Cell Mol Med. (2023) 27:939–49. doi: 10.1111/jcmm.17700. PMID: 36919822 PMC10064033

[15] Huang J Liang L Jiang S Liu Y He H Sun X . BDH1-mediated LRRC31 regulation dependent on histone lysine β-hydroxybutyrylation to promote lung adenocarcinoma progression. Medcomm (2020). (2023) 4:e449. doi: 10.1002/mco2.449. PMID: 38098610 PMC10719427

[16] Persson B Kallberg Y . Classification and nomenclature of the superfamily of short-chain dehydrogenases/reductases (SDRs). Chem Biol Interact. (2013) 202:111–5. doi: 10.1016/j.cbi.2012.11.009. PMID: 23200746

[17] Persson B Kallberg Y Bray JE Bruford E Dellaporta SL Favia AD . The SDR (short-chain dehydrogenase/reductase and related enzymes) nomenclature initiative. Chem Biol Interact. (2009) 178:94–8. doi: 10.1016/j.cbi.2008.10.040. PMID: 19027726 PMC2896744

[18] Persson B Krook M Jörnvall H . Characteristics of short-chain alcohol dehydrogenases and related enzymes. Eur J Biochem. (1991) 200:537–43. doi: 10.1111/j.1432-1033.1991.tb16215.x. PMID: 1889416

[19] Marks AR McIntyre JO Duncan TM Erdjument-Bromage H Tempst P Fleischer S . Molecular cloning and characterization of (R)-3-hydroxybutyrate dehydrogenase from human heart. J Biol Chem. (1992) 267:15459–63. doi: 10.1016/s0021-9258(19)49556-2 1639787

[20] Cheng C Geng F Cheng X Guo D . Lipid metabolism reprogramming and its potential targets in cancer. Cancer Commun (Lond). (2018) 38:27. doi: 10.1186/s40880-018-0301-4. PMID: 29784041 PMC5993136

[21] Li Z Sun C Qin Z . Metabolic reprogramming of cancer-associated fibroblasts and its effect on cancer cell reprogramming. Theranostics. (2021) 11:8322–36. doi: 10.7150/thno.62378. PMID: 34373744 PMC8343997

[22] Xia L Oyang L Lin J Tan S Han Y Wu N . The cancer metabolic reprogramming and immune response. Mol Cancer. (2021) 20:28. doi: 10.1186/s12943-021-01316-8. PMID: 33546704 PMC7863491

[23] Otsuka H Kimura T Ago Y Nakama M Aoyama Y Abdelkreem E . Deficiency of 3-hydroxybutyrate dehydrogenase (BDH1) in mice causes low ketone body levels and fatty liver during fasting. J Inherit Metab Dis. (2020) 43:960–8. doi: 10.1002/jimd.12243. PMID: 32279332

[24] Sun S Guo L Zhu H . BDH1 drives ovarian cancer progression by regulating cell cycling and stemness maintenance through Wnt/β-catenin signaling. Int J Biol Markers. (2026), 3936155261435599. doi: 10.1177/03936155261435599. PMID: 41885548

[25] Wei X Zhang C Zheng X Pan K Ren X Lou X . β-hydroxybutyrate dehydrogenase promotes pancreatic cancer cell proliferation through regulation of the NAD(+)/NADH balance and mitochondrial acetylation. J Biol Chem. (2025) 301:110636. doi: 10.1016/j.jbc.2025.110636. PMID: 40885386 PMC12494558

[26] Xiang J Su R Wu S Zhou L . Construction of a prognostic signature for serous ovarian cancer based on lactate metabolism-related genes. Front Oncol. (2022) 12:967342. doi: 10.3389/fonc.2022.967342. PMID: 36185201 PMC9520471

[27] Shukla SK Gebregiworgis T Purohit V Chaika NV Gunda V Radhakrishnan P . Metabolic reprogramming induced by ketone bodies diminishes pancreatic cancer cachexia. Cancer Metab. (2014) 2:18. doi: 10.1186/2049-3002-2-18. PMID: 25228990 PMC4165433

[28] Schaff LR Mellinghoff IK . Glioblastoma and other primary brain Malignancies in adults: a review. Jama. (2023) 329:574–87. doi: 10.1001/jama.2023.0023. PMID: 36809318 PMC11445779

[29] Maurer GD Brucker DP Bähr O Harter PN Hattingen E Walenta S . Differential utilization of ketone bodies by neurons and glioma cell lines: a rationale for ketogenic diet as experimental glioma therapy. BMC Cancer. (2011) 11:315. doi: 10.1186/1471-2407-11-315. PMID: 21791085 PMC3199865

[30] Vallejo FA Shah SS de Cordoba N Walters WM Prince J Khatib Z . The contribution of ketone bodies to glycolytic inhibition for the treatment of adult and pediatric glioblastoma. J Neuro-Oncol. (2020) 147:317–26. doi: 10.1007/s11060-020-03431-w. PMID: 32096068

[31] Fakhri N Chad MA Lahkim M Houari A Dehbi H Belmouden A . Risk factors for breast cancer in women: an update review. Med Oncol. (2022) 39:197. doi: 10.1007/s12032-022-01804-x. PMID: 36071255

[32] Hong JH Ko YH Kang K . RNA variant identification discrepancy among splice-aware alignment algorithms. PloS One. (2018) 13:e0201822. doi: 10.1371/journal.pone.0201822. PMID: 30071094 PMC6072070

[33] Martinez-Outschoorn UE Lin Z Whitaker-Menezes D Howell A Lisanti MP Sotgia F . Ketone bodies and two-compartment tumor metabolism: stromal ketone production fuels mitochondrial biogenesis in epithelial cancer cells. Cell Cycle. (2012) 11:3956–63. doi: 10.4161/cc.22136. PMID: 23082721 PMC3507491

[34] Kratzer TB Mazzitelli N Star J Dahut WL Jemal A Siegel RL . Prostate cancer statistics, 2025. CA Cancer J Clin. (2025) 75:485–97. doi: 10.3322/caac.70028. PMID: 40892160 PMC12593258

[35] Sekhoacha M Riet K Motloung P Gumenku L Adegoke A Mashele S . Prostate cancer review: genetics, diagnosis, treatment options, and alternative approaches. Molecules. (2022) 27(17):5730. doi: 10.3390/molecules27175730. PMID: 36080493 PMC9457814

[36] Wasim S Lee SY Kim J . Complexities of prostate cancer. Int J Mol Sci. (2022) 23(22):14257. doi: 10.3390/ijms232214257. PMID: 36430730 PMC9696501

[37] Saraon P Cretu D Musrap N Karagiannis GS Batruch I Drabovich AP . Quantitative proteomics reveals that enzymes of the ketogenic pathway are associated with prostate cancer progression. Mol Cell Proteomics. (2013) 12:1589–601. doi: 10.1074/mcp.M112.023887. PMID: 23443136 PMC3675816

[38] Chen XB Yang ZH Ling WJ Feng QJ Lu ZY . Global burden of leukemia in women of child-bearing age, 1990 to 2021: an update from the Global Burden of Disease Study 2021. Med (Baltimore). (2026) 105:e47217. doi: 10.1097/MD.0000000000047217. PMID: 41578459 PMC12851764

[39] GBD 2023 MASLD Collaborators . Global burden of metabolic dysfunction-associated steatotic liver disease, 1990-2023, and projections to 2050: a systematic analysis for the Global Burden of Disease Study 2023. Lancet Gastroenterol Hepatol. (2026) 11(6):463–494. doi: 10.1016/S2468-1253(26)00011-7. PMID: 41990758

[40] Toh MR Wong EYT Wong SH Ng AWT Loo LH Chow PK . Global epidemiology and genetics of hepatocellular carcinoma. Gastroenterology. (2023) 164:766–82. doi: 10.1053/j.gastro.2023.01.033. PMID: 36738977

[41] Huo J Wu L Zang Y . Development and validation of a metabolic-related prognostic model for hepatocellular carcinoma. J Clin Transl Hepatol. (2021) 9:169–79. doi: 10.14218/jcth.2020.00114. PMID: 34007798 PMC8111106

[42] Sabari BR Zhang D Allis CD Zhao Y . Metabolic regulation of gene expression through histone acylations. Nat Rev Mol Cell Biol. (2017) 18:90–101. doi: 10.1038/nrm.2016.140. PMID: 27924077 PMC5320945

[43] Thai AA Solomon BJ Sequist LV Gainor JF Heist RS . Lung cancer. Lancet. (2021) 398:535–54. doi: 10.1016/s0140-6736(21)00312-3. PMID: 34273294

[44] Zhang J Jia PP Liu QL Cong MH Gao Y Shi HP . Low ketolytic enzyme levels in tumors predict ketogenic diet responses in cancer cell lines *in vitro* and *in vivo*. J Lipid Res. (2018) 59:625–34. doi: 10.1194/jlr.M082040. PMID: 29414764 PMC5880499

[45] Saks V Schlattner U Tokarska-Schlattner M Wallimann T Bagur R Zorman S . Molecular system bioenergetics—integration of our knowledge of old and new pathways with the control of cellular energetics. FEBS J. (2016) 283:54–73. doi: 10.1111/febs.13566. PMID: 26417966

[46] Zuo P Wei Y Ma X Wu T . Global, regional, and national burdens of lip and oral cavity cancer in the adolescents and young adults from 1990 to 2021 and its predictions. Med (Baltimore). (2025) 104:e45601. doi: 10.1097/MD.0000000000045601. PMID: 41248696 PMC12599713

[47] Yousefi M Karimi A Goudarzi A . The association of ketolytic enzymes gene expression levels with mitochondrial activity and content in oral squamous cell carcinoma. Asian Pac J Cancer Prev. (2022) 23:3953–8. doi: 10.31557/apjcp.2022.23.11.3953. PMID: 36444610 PMC9930959

[48] Chattopadhyay T Gupta P Nayak R Mallick B . Genome-wide profiling of dysregulated piRNAs and their target genes implicated in oncogenicity of tongue squamous cell carcinoma. Gene. (2023) 849:146919. doi: 10.1016/j.gene.2022.146919. PMID: 36179965

